# Integrated Assessment of PAH Contamination in the Czech Rivers Using a Combination of Chemical and Biological Monitoring

**DOI:** 10.1155/2014/918097

**Published:** 2014-01-28

**Authors:** Jana Blahova, Lenka Divisova, Vit Kodes, Drahomira Leontovycova, Samuel Mach, Tomas Ocelka, Zdenka Svobodova

**Affiliations:** ^1^Department of Veterinary Public Health and Animal Welfare, University of Veterinary and Pharmaceutical Sciences Brno, Palackého tř. 1/3, 612 42 Brno, Czech Republic; ^2^Czech Hydrometeorological Institute, Na Šabatce 17, 143 06, Prague 4, Czech Republic; ^3^Institute of Public Health Ostrava, Partyzánské nám. 7, 702 00 Ostrava, Czech Republic

## Abstract

This study investigated polycyclic aromatic hydrocarbons (PAH) pollution of selected rivers in the Czech Republic. Integrated evaluation was carried out using combination of chemical and biological monitoring, in which we measured content of 1-hydroxypyrene (1-OHP) in chub bile and priority PAH in water samples obtained by exposing the semipermeable membrane devices at each location. The concentrations of 1-OHP in bile samples and sum of priority PAH in water sampler ranged from 6.8 ng mg protein^−1^ to 106.6 ng mg protein^−1^ and from 5.2 ng L^−1^ to 173.9 ng L^−1^, respectively. The highest levels of biliary metabolite and PAH in water were measured at the Odra River (the Bohumín site), which is located in relatively heavily industrialized and polluted region. Statistically significant positive correlation between biliary 1-OHP and sum of PAH in water was also obtained (*P* < 0.01, *r*_*s*_ = 0.806).

## 1. Introduction

Polycyclic aromatic hydrocarbons (PAH) are a large group of important environmental organic pollutants which occur ubiquitously and are typically more concentrated near urban center and their concentrations correlate with shipping traffic or combustion of fossil fuel. Because of their mutagenic and/or procarcinogenic properties to human and animals they are routinely monitored in different matrices (air, water, sediment, animal tissues, etc.) [1]. The US Environmental Protection Agency has promulgated sixteen unsubstituted PAH (acenaphthylene, acenaphthene, anthracene, benzo[*a*]anthracene, benzo[*a*]pyrene, benzo[*b*]fluoranthene, benzo[*k*]fluoranthene, benzo[*ghi*]perylen, chrysene, dibenzo[*ah*]anthracene, fluoranthene, fluorene, indeno[*1,23-cd*]pyrene, naphthalene, phenanthrene, and pyrene) as priority pollutants. The monitoring of sum of these priority pollutants is often used for assessment of PAH occurrence in the ecosystem [2].

There are many sources of PAH to aquatic ecosystem that include wastewater from industrial activities, urban and rural runoff, production of fossil fuels, petroleum spilling during transportation, leachate from solid waste disposal dumps, or atmospheric deposition [3–6]. Polycyclic aromatic hydrocarbons entering aquatic environment, due to their hydrophobicity, rapidly become associated with suspended particles and sediment. Sorption and retention capacity depends on physicochemical properties of sediment and suspended particles such as grain size or content of organic matter [1].

Polycyclic aromatic hydrocarbons enter aquatic animals especially via gastrointestinal tract (food, water, and sediment) or via passive diffusion through the gills and body surface [3, 7]. In most of aquatic animals, they are rapidly metabolized into water soluble products and eliminated from organism. The bioaccumulation ability of PAH is very variable and was observed especially at the first trophic levels [4]. In fish, PAH are biotransformed predominantly by liver through oxidation (phase I) and conjugation (phase II) reactions to hydrophilic metabolites which are subsequently deposited in the gall bladder and excreted from the organism through bile [8]. Many field and experimental studies have demonstrated that biliary PAH metabolites are sensitive measures and correlate well with the PAH exposure in fish [9–11]. Many studies have identified 1-hydroxypyrene (1-OHP) as one of the most abundant compounds present in fish bile and this metabolite is regarded as the best general indicator of short-term PAH exposure in fish [12]. A range of analytical technics is available for the determination of fish biliary PAH metabolites. The most rapid screening assay is simple fluorescence assay (fixed fluorescence detection or synchronous fluorescence spectrometry). This method is suitable for screening fish bile samples *in situ* at field locations, where relevant PAH pollution incident has occurred (e.g., oil spill). However, fluorescence techniques are not able to quantify specific metabolites in complex bile matrix. In order to provide selective and quantitative determination of biliary metabolites, chromatographic separation is required. Prior to the chromatographic separation, the bile sample is typically subjected to various forms of pretreatment (enzymatic hydrolysis, solid phase extraction, or derivatization) [12].

The aim of this field study was to investigate the PAH contamination at outlet sites of river in the Czech Republic using fish biliary metabolite (1-OHP). The values of this biochemical marker were correlated with the PAH content in water samples obtained by exposing the semipermeable membrane devices (SPMD) at each location for four weeks.

## 2. Materials and Methods

### 2.1. Site Description and Animals Sampling

**Figure 1:**
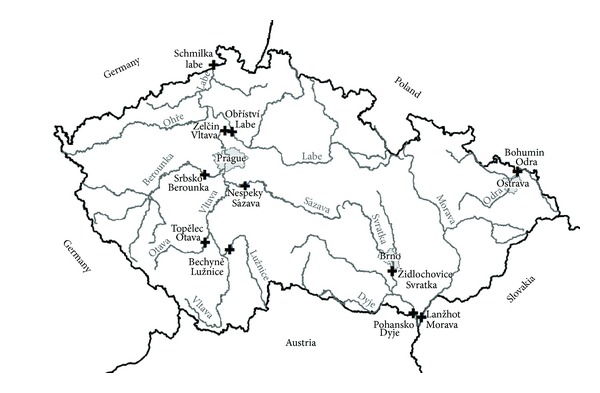
Study area in the Czech Republic.

The field study was conducted at eleven sites on ten major rivers in the Czech Republic (Figure 1) in 2011. The locations were the Lužnice site (the Bechyně River, river km 11), the Topělec site (the Otava River, river km 20), the Nespeky site (the Sázava River, river km 27.5 km), the Srbsko site (the Berounka River, river km 29), the Zelčín site (the Vltava River, river km 5), the Obříství site (the Labe River, river km 122), the Schmilka site (the Labe River, river km 21), the Židlochovice site (the Svratka River, river km 23), the Pohansko site (the Dyje River, river km 16), the Lanžhot site (the Morava River, river km 9.5), and the Bohumín site (the Odra River, river km 9). The chub (*Leuciscus cephalus *L.) was selected as the most suitable indicator species, because it is a common freshwater cyprinid fish that inhabits both clean and polluted rivers. Fish were captured by electrofishing and weighed and their scales collected for age determination. Bile was drawn by needle through the exposed gall bladder; samples were frozen in liquid nitrogen and taken to the laboratory, where they were stored at −85°C for further processing. One passive sampler, a semipermeable membrane device, was placed at each site for four weeks. Semipermeable membrane device samplers consist of a horizontal thin-walled tub from nonporous low density polyethylene, filled with synthetic lipid (triolein) and they were commercially manufactured (Exposmeter AB, Sweden), with their quality assurance and quality control which made it possible to compare results.

### 2.2. Determination of 1-OHP

Levels of biliary 1-OHP were determined according to the method described previously by Blahova et al. (2008) [13]. Bile samples were deconjugated with an enzyme mixture of glucuronidase and arylsulphatase (37°C, 1 hour) and purified on solid phase extraction column (LiChrolut EN, Merck). The samples were eluted from the cartridges with acetone, vaporized in a nitrogen atmosphere, and resuspended in 300 *μ*L of methanol. Aliquot of the extract was injected onto Polaris column (C18-A, 3 *μ*, 150 × 4.6 mm) in a HPLC system with fluorescence detector (*λ*_ex_ = 364 nm, *λ*_em_ = 384 nm) (Waters, USA) and separation was performed using an acetonitrile : water mobile phase with the linear gradient as follows: *t* = 0 min: 65% acetonitrile, *t* = 5 min: 70% acetonitrile, *t* = 10 min: 80% acetonitrile, and *t* = 12 min: 65% acetonitrile. Content of 1-OHP was normalized and expressed as ng of metabolite per mg of total protein. Total biliary protein was quantified by a spectrophotometric method using bicinchoninic acid and bovine serum albumin as a standard.

### 2.3. Determination of Priority PAH in Passive Sampler

After exposure, the membranes were cleaned with brief rinses of deionized water to remove superficial deposits and placed on ice for transport to the laboratory at −18°C until chromatographic analyses. Before chromatography analyses, membranes were rinsed by acetone and hexane prior to extraction by dialysis against hexane. The low molecular weight PAH were analyzed using gas chromatography with mass spectrometry and high performance liquid chromatography and fluorescence detection was used for the identification and quantification of high molecular weight PAH [14, 15]. The analyses of PAH were carried out by laboratories of the Centre of Hygienic Laboratories Ostrava, which are accredited by the Czech Accreditation Institute.

### 2.4. Statistical Analysis

Results of biliary 1-OHP were tested for normal distribution using the Shapiro-Wilk test. Data were log-transformed and a one way analysis of variance (ANOVA) was applied to test the differences in biliary metabolite among sites. Individual differences between the means were tested successively using Tukey-HSD test. The Spearman rank correlation coefficient (*r*_*s*_) was used for determination of relationship between biliary 1-OHP and sum of priority PAH in passive samplers. Significance was accepted at *P* < 0.05. The statistical analysis was performed using Unistat 5.6. software.

## 3. Results

The results of concentration of sixteen priority PAH in water samples obtained by exposing the SPMD at sampling sites are summarized in Table 1. Because of technical problem, SPMD from the Schmilka site (the Labe River) did not produce sample. The highest content was obtained at the Bohumín site, the Odra River (173.9 ng L^−1^); this value was more than thirty times higher than that at the location Obříství, the Labe River (5.2 ng L^−1^), where the lowest concentration of PAH was found. The levels of individual PAH at each location varied between not quantified and 89 ng L^−1^. The three and four rings PAH (namely, acenaphthene, phenanthrene, fluorene, fluoranthene, and pyrene) represented the most prevalent PAH. The least occurring PAH was dibenzo[*ah*]anthracene; it was detectable only at two sites.

In time of SPMD exposing, indicator fish were caught and bile samples were collected for determination of 1-OHP at all sites. The numbers of individuals, length, and weight of chubs for each location are given in Table 2.

**Figure 2:**
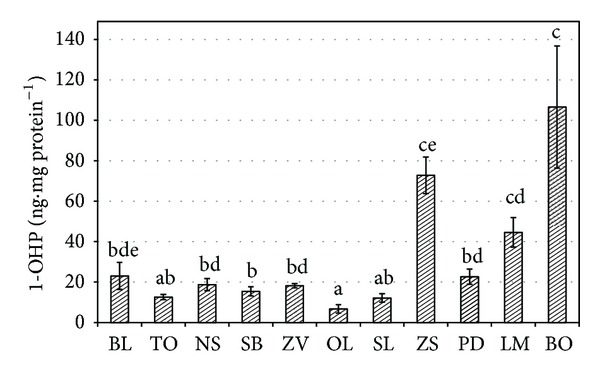
Content (mean ± standard error of mean) of 1-OHP in bile samples from fish at monitored sites (BL: Berounka site/Lužnice River; TO: Topělec site/Otava River; NS: Nespeky site/Sázava River; SB: Srbsko site/Berounka River; ZV: Zelčín site/Vltava River; OL: Obříství site/Labe River; SL: Schmilka site/Labe River; ZS: Židlochovice site/Svratka River; PD: Pohansko site/Dyje River; LM: Lanžhot site/Morava River; BO: Bohumín site/Odra River). Significant differences (*P* < 0.05) are indicated by different alphabetic superscripts.

The results of biliary 1-OHP are presented in Figure 2. Because of differences in density of individual samples, data were normalized to the protein content. 1-Hydroxypyrene was detected in all samples, the lowest individual value was found at the Obříství site, the Labe River (2.2 ng mg protein^−1^), and the highest one at the Bohumín site, the Odra River (165.2 ng mg protein^−1^). The same trend was also recorded in the average values at the monitored locations. The highest mean value was observed at the Bohumín site—the Odra River (106.6 ± 30.2 ng mg protein^−1^), and it was significantly (*P* < 0.05) higher than those obtained at the Obříství site—the Labe River (6.8 ± 2.1 ng mg protein^−1^); the Schmilka site—the Labe River (12.1 ± 2.1 ng mg protein^−1^); the Topělec site—the Otava River (12.6 ± 1.3 ng mg protein^−1^); the Srbsko site—the Berounka River (15.4 ± 2.2 ng mg protein^−1^); the Zelčín site—the Vltava River (18.2 ± 1.0 ng mg protein^−1^); the Nespeky site, the Sázava River (18.8 ± 2.3 ng mg protein^−1^); the Pohansko site, the Dyje River (22.7 ± 3.7 ng mg protein^−1^); and the Bechyně site, the Lužnice River (23.0 ± 6.7 ng mg protein^−1^). No significant differences were found between the Bohumín site, the Odra River and the Lanžhot site, the Morava River (44.6 ± 7.3 ng mg protein^−1^) and the Židlochovice site, the Svratka River (72.8 ± 9.0 ng mg protein^−1^).

Highly statistically significant positive correlation (*P* < 0.01) between biliary 1-OHP and sum of priority PAH was also obtained (*r*_*s*_ = 0.806).

## 4. Discussion

Chemical monitoring such as measurement of pollutants content in the aquatic environment (water column, sediment, etc.) and residue levels in the aquatic organisms is the most widely used method for assessment of aquatic contamination. Unfortunately, the presence of contaminants does not, by itself, indicate adverse effects on the organisms; it is appropriate to combine chemical monitoring with biological assessment. During biological assessment the responses of aquatic organisms to pollution stress can be evaluated by measurement of biochemical markers levels. Biomarker is defined as a change in a biological response which can be related to exposure to or toxic effects of environmental chemicals [7]. Many experimental studies demonstrated that suitable fish biomarkers for PAH exposure may be (i) concentration of biliary PAH metabolites, (ii) ethoxyresorufin-*O*-deethylase activity in liver estimating cytochrome P4501A induction, or (iii) formation of DNA adducts in liver and blood [7, 16]. Responses in these biochemical markers in fish have been correlated with more adverse effects, such as liver neoplasia [17]. In our study we used the combination of chemical and biological monitoring for complex assessment of PAH contamination in the selected major rivers in the Czech Republic. From the biomarkers we have chosen determination of PAH metabolite which was analyzed in the chub bile. The obtained results of biliary metabolite were correlated with the levels of priority PAH in abiotic matrix. Concentration of polycyclic aromatic hydrocarbons was monitored in water samples which were obtained using modern technology of passive sampling. This technology has potential to become a reliable, robust, and cost effective tool in monitoring of aquatic contamination. In the passive sampling, analyte concentrations are integrated over the sampling time and this method is less sensitive to accidental and extreme variations of pollutant concentration. For hydrophobic pollutants, such as PAH, the sampler SPMD is most widely used [18].

Based on the previous studies [3, 11, 19], we chose only one PAH metabolite which we analyzed in the chub bile. In general, many studies have identified 1-OHP as one of the most abundant compounds present in fish bile and this metabolite is regarded as the best general indicator of PAH exposure in fish [7, 10, 11, 13]. Ruddock et al. [9] documented that 1-OHP accounts for up to 76% of all PAH metabolites in fish bile. 1-Hydroxypyrene is the main degradation product of pyrene—a widespread PAH generated by many pyrolytic and petrogenic industrial processes [4, 7, 10]. Use of biliary 1-OHP for assessment of PAH contamination has been previously performed in several marine and fresh fish species, such as chub (*Leuciscus cephalus* L.) [11, 13, 19], nile tilapia (*Oreochromis niloticus*) [8], European eel (*Anguilla anguilla*) [9, 20], Atlantic cod (*Gadus morhua*) [16], English sole (*Pleuronectes vetulus*) [17], roach (*Rutilus rutilus *L.), [19] or brown trout (*Salmo trutta m. fario *L.) [19]. Some studies also recommended that biliary PAH metabolite contents should be normalized to biliverdin or protein concentration in the bile due to a different feeding status of some fish and due to variable water level in the bile [8, 9].

In this study we detected 1-OHP in all bile samples, the individual results were very variable and ranged from 2.2 ng mg protein^−1^ to 165.2 ng mg protein^−1^. The mean content of 1-OHP varied significantly between several sites, with the highest mean concentration in bile from the Odra River at Bohumín site. This mean concentration was more than fifteen times higher than that at the Obříství site, the Labe River, where the lowest content of 1-OHP was detected. Results of biliary metabolite correspond with concentrations of priority PAH in water samples, because the highest PAH contamination was also observed at the Bohumín site, Odra River, and the lowest one at the Obříství site—the Labe River. The PAH levels at the Bohumín site—Odra River were more than thirty times higher than those at the Obříství site—the Labe River. The Odra River flows through a heavily industrialized (coal mining, heat, and power generating plants) and urbanized region in the north of the Czech Republic. Several studies confirmed that the occurrence of various pollutants including PAH arises from the intensive anthropogenic activities in this region [21–23]. Blahova et al. [11] monitored the levels of PAH pollution in selected Czech rivers and the highest content of priority PAH in sediment samples was found at the Odra River (sampling site at the Bohumín) which is in agreement with results from our study. Wolska and Namieśnik [21] studied distribution of selected organic pollutants in the Odra River in Poland. The PAH concentrations detected in water samples from the Odra River and its tributaries ranged from detection limit (1.0 ng L^−1^) to 1,500 ng L^−1^. The maximum detected value is several times higher than we present in our study. The differences may be caused by different sampling methods, because in our study we used continuous sampling using SPMD, while in the study by Wolska and Namieśnik [21] the conventional sampling technique was used. Conventional water sampling has several limitations; especially water samples reflect residue composition only at the moment of sampling and may fail to detect episodic contamination [18]. In recent years, the use of modern techniques of passive sampling for monitoring of aquatic environment contamination is still expanding and it is recommended for the biomonitoring studies. Several studies applied SPMD sampling for evaluation of PAH content in aquatic environment such as rivers, lakes, or reservoirs [19, 24–26].

The results from our study show strong positive and significant correlation (*P* < 0.01, *r*_*s*_ = 0.806) between biliary 1-OHP and PAH in water samples. Similar findings also document positive relationship between biliary metabolites and content of PAH in both water [11, 13] and sediment samples [19]. Hosnedl et al. [27] documented positive correlation between 1-OHP in fish bile and concentrations of priority PAH in sediment. For both species, chub (*Leuciscus cephalus *L.) and bream (*Abramis brama*), the correlation coefficients were 0.81 and 0.76, respectively. They also confirmed relationship between 1-OHP and content of pyrene in sediment (*r*_*s*_ = 0.78 and 0.77, resp.). This finding indirectly confirms the assumption of an appropriate relation between pyrene concentration and sum of priority PAH occurring in particular environmental compartment.

## 5. Conclusion

In conclusion, the results of the presented study indicate that the use of 1-OHP as biomarker in fish is useful tool for monitoring of PAH contamination in the aquatic ecosystem. Determination of this metabolite is also frequently used as a useful biochemical marker for assessment of human exposure to PAH, especially in smokers, drivers, or workers in aluminum plants, oil refineries, and coal-burning facilities. The analyses of 1-OHP in human are provided in urine samples [28, 29].

## Figures and Tables

**Table 1:** Content of priority PAH in SPMD samples from sampling sites.

Locality	Sum of priority PAH (ng L^−1^)
Bechyně (Lužnice River)	6.6
Topělec (Otava River)	10.6
Nespeky (Sázava River)	5.3
Srbsko (Berounka River)	5.5
Zelčín (Vltava River)	5.4
Obříství (Labe River)	5.2
Schmilka (Labe River)	n.m.
Židlochovice (Svratka River)	14.2
Pohansko (Dyje River)	6.3
Lanžhot (Morava River)	14.8
Bohumín (Odra River)	173.9

n.m: sample was not measured because of the technical problem.

**Table 2:** Biometric** c**haracteristics of male chub from sampling sites.

Locality	Fish (*n*)	Mean age (min–max) (years)	Mean weight ± SD (g)
Bechyně (Lužnice River)	2	3.5 (3-4)	142.5 ± 74.2
Topělec (Otava River)	6	3.0 (3-3)	84.2 ± 7.4
Nespeky (Sázava River)	6	3.0 (3-3)	93.3 ± 7.5
Srbsko (Berounka River)	6	4.0 (4-4)	174.2 ± 18.6
Zelčín (Vltava River)	6	5.7 (5-6)	393.3 ± 79.6
Obříství (Labe River)	5	4.0 (4-4)	196.3 ± 45.9
Schmilka (Labe River)	6	4.8 (4-5)	447.5 ± 142.9
Židlochovice (Svratka River)	6	4.2 (4-5)	225.8 ± 28.5
Pohansko (Dyje River)	6	5.2 (4–6)	530.0 ± 160.8
Lanžhot (Morava River)	4	3.7 (3-4)	165.0 ± 65.4
Bohumín (Odra River)	3	3.3 (3-4)	91.7 ± 30.1

SD: standard deviation.
